# Pharmacological targeting of NF-*κ*B potentiates the effect of the topoisomerase inhibitor CPT-11 on colon cancer cells

**DOI:** 10.1038/sj.bjc.6604082

**Published:** 2008-01-08

**Authors:** P Lagadec, E Griessinger, M P Nawrot, N Fenouille, P Colosetti, V Imbert, M Mari, P Hofman, D Czerucka, D Rousseau, E Berard, M Dreano, J F Peyron

**Affiliations:** 1 INSERM U526, Nice F-06000, France; 2 Faculté de Médecine Pasteur (IFR50), Université Nice Sophia-Antipolis, Nice F-06107, France; 3 INSERM ERI 21, Nice F-06000, France; 4 INSERM U721, Nice F-06000, France; 5 CHU de Nice, Service de Pédiatrie, Hôpital de l'Archet II, Nice F-06200, France; 6 Merck-Serono International S.A., Geneva, Switzerland

**Keywords:** CPT-11, chemoresitance, NF-*κ*B, colorectal cancer, *in vivo*

## Abstract

NF-*κ*B interferes with the effect of most anti-cancer drugs through induction of anti-apoptotic genes. Targeting NF-*κ*B is therefore expected to potentiate conventional treatments in adjuvant strategies. Here we used a pharmacological inhibitor of the IKK2 kinase (AS602868) to block NF-*κ*B activation. In human colon cancer cells, inhibition of NF-*κ*B using 10 *μ*M AS602868 induced a 30–50% growth inhibitory effect and strongly enhanced the action of SN-38, the topoisomerase I inhibitor and CPT-11 active metabolite. AS602868 also potentiated the cytotoxic effect of two other antineoplasic drugs: 5-fluorouracil and etoposide. In xenografts experiments, inhibition of NF-*κ*B potentiated the antitumoural effect of CPT-11 in a dose-dependent manner. Eighty-five and 75% decreases in tumour size were observed when mice were treated with, respectively, 20 or 5 mg kg^−1^ AS602868 associated with 30 mg kg^−1^ CPT-11 compared to 47% with CPT-11 alone. *Ex vivo* tumour analyses as well as *in vitro* studies showed that AS602868 impaired CPT-11-induced NF-*κ*B activation, and enhanced tumour cell cycle arrest and apoptosis. AS602868 also enhanced the apoptotic potential of TNF*α* on HT-29 cells. This study is the first demonstration that a pharmacological inhibitor of the IKK2 kinase can potentiate the therapeutic efficiency of antineoplasic drugs on solid tumours.

Colorectal cancer (CRC) is the third commonest malignancy worldwide with 954 000 new cases and 492 000 deaths in 2000 (Davies *et al*, 2005). These outcomes are largely due to the poor clinical response of CRC to conventional drugs. CPT-11 (irinotecan) and its active metabolite, SN-38, are topoisomerase I inhibitors that have shown efficacy in the treatment of advanced and/or metastatic CRC (Wasserman *et al*, 2001). However, despite the initial response, most patients treated with CPT-11 become resistant and exhibit tumour progression (Calvo *et al*, 2002). CPT-11 treatment has been shown to activate NF-*κ*B (Xu and Villalona-Calero, 2002; Janssens and Tschopp, 2006), which could be a potential resistance mechanism in malignant cells (Wang *et al*, 1998; Baldwin, 2001; Nakanishi and Toi, 2005). Thus, reducing NF-*κ*B-mediated activation may help prevent CPT-11-induced resistance to cell killing.

NF-*κ*B complexes are composed of a variety of homo- or heterodimers formed by five components: p50, p52, p65 (-RelA), RelB, and c-Rel subunits. The p50–p65 complexes are the best-characterized and most abundant dimers. In the absence of stimulation, NF-*κ*B is sequestered in the cytoplasm of most cells, by binding to I*κ*B inhibitory subunits. Upon stimulation, I*κ*B molecules are phosphorylated by the specific kinases IKK(I*κ*B kinase)1/*α* and IKK2/*β*, which together with NEMO (NF-*κ*B Essential Modulator) /IKK*γ* form the IKK complex that integrates signals for NF-*κ*B activation. Serine phosphorylation is followed by polyubiquitination, and subsequent degradation of I*κ*B by the proteasome reviewed in Karin (1999). Then, NF-*κ*B translocates into the nucleus where it controls the transcription of numerous genes. Mechanisms by which topoisomerase-targeting drugs induce I*κ*B degradation to activate the NF-*κ*B pathway have to be elucidated. In response to DNA damage, NEMO appears to translocate to the nucleus and undergo a series of post-translational modifications. In the nucleus, NEMO establishes a complex with p53-inducible protein with a death domain and receptor interacting protein 1, allowing NEMO sumoylation (Janssens and Tschopp, 2006). Then, sumo-NEMO is recognized and phosphorylated by ATM (Ataxia Telangiectasia Mutated), tagged by ubiquitination, which induces its release from ATM and its cytoplasmic translocation allowing NF-*κ*B activation (Wu *et al*, 2006). Thus, NEMO provides a means to link nuclear DNA damage to the activation of the cytoplasmic IKK complex (Huang *et al*, 2003). Once activated, NF-*κ*B promotes cell survival through expression of genes coding for antiapoptotic proteins (c-IAP1, c-IAP2, bfl-1, and Bcl-xl) and supports resistance of tumour cell to treatments by inducing the expression of the multidrug resistance proteins (Pahl, 1999). Furthermore, NF-*κ*B could largely participate to the tumorigenic process through expression of genes coding for growth factors and cell cycle regulators (Van Antwerp *et al*, 1996; Wang *et al*, 1996) as well as it could promote metastasis through induction of extracellular matrix-degrading enzymes and angiogenesis through vascular endothelial growth factor expression (Yu *et al*, 2004). The inhibition of NF-*κ*B could therefore affect tumour cells at different steps of their pathological process.

The aim of our study was to evaluate the effect of inhibiting NF-*κ*B to potentiate the action of the topoisomerase poison CPT-11 in colon cancer cells. We used a pharmacological inhibitor of the IKK2 kinase (AS602868) that was previously shown to reveal the apoptotic potential of TNF-*α* in Jurkat cells (Frelin *et al*, 2003) and to induced apoptosis of primary human acute myeloid leukaemia cells (Frelin *et al*, 2005). We show that both *in vitro* and *in vivo* in HT-29 colon s.c. xenografts AS602868 potentiated antitumour CPT-11 effectiveness by increasing CPT-11-induced apoptosis of HT-29 tumour cells. This effect was associated with decreased expression of antiapoptotic genes and a stimulation of CPT-11 antiproliferative actions. The antitumoural effect of AS602868 could also be due to its capacity to induce apoptosis of HT-29 cells in the presence of TNF*α* whose intratumoural concentration was increased upon CPT-11 treatment.

## MATERIALS AND METHODS

### Drugs and antibodies

AS602868 is an anilino-pyrimidine derivative and ATP competitor selected for its inhibitory effect *in vitro* on IKKee, a constitutively active version of IKK2. The compound is covered by the patent application PCT WO 02/46171. AS602868 has an *in vitro* inhibitory concentration of 50% (IC_50_) of 60 nM towards purified IKK2 and no effect on IKK1 (IC_50_=14 *μ*M) or on a large panel of recombinant kinases. It has some inhibitory effect on JNK2 (IC_50_=600 nM). AS602868 in sterile cyclodextrin solution was supplied by Merck-Serono International SA (Geneva, Switzerland). CPT-11 was a gift from Dr Pierre-Alain Vitte (Serono Pharmaceutical Research Institute, Geneva, Switzerland). The pan caspase inhibitor z-VAD^fmk^ (R&D Systems, Abington, UK) and SN-38 (a kind gift from Dr JL Fischel, Antoine Lacassagne Oncology Center, Nice, France) were prepared in DMSO and stock solutions were stored at −20°C. Recombinant hTNF*α* was from PeproTech (Rocky Hill, NJ, USA). Anti-Parp-*α* and anti-phospho I*κ*B was purchased from Cell Signaling (Beverly, MA, USA); anti-HSP60, anti-p65 p50, anti-I*κ*B, and anti-TNF*α* from Santa Cruz Biotechnology (Santa Cruz, CA, USA); anti-caspases 3, 8, and 9 from Medical & Biological Laboratories (Woburn, MA, USA); and anti-Ki-67 from DAKOCytomation (Glostrup, Denmark).

### Cell lines and cell drug treatments

The human colon cancer cell lines HT-29, SW-480, and SW-620 were obtained from the ATCC (Bethesda, MD, USA). Aliquots of 5 × 10^6^ viable cells in 10 ml of DMEM medium containing 10% fetal calf serum were plated into tissue culture dishes (100 mm diameter) for 24 h, then stimulated for 72 h before harvesting.

### Xenograft growth assay

Animal experiments were performed in accordance with the regulations of our institution's ethics commission and with the United Kingdom Co-ordinating Committee on Cancer Research Guidelines (1998). Forty-five NMRI female nude mice (6–8 weeks of age) were inoculated s.c. with 1 × 10^6^ tumour cells. Mice were then dispatched into nine groups of 5. Treatments lasted 10 weeks and consisted of five orally administrations of AS602868 (5 or 20 mg kg^−1^), 5 days a week. CPT-11 (10 or 30 mg kg^−1^) was administered i.p. twice a week. In combination treatments, AS602868 was given 4 h before CPT-11 injections. Mice from control group were administered with AS602868 vehicle (cyclodextrin). Tumours were measured once a week with a caliper and their volumes were calculated by the formula: (*a* × *b*^2^)/2, where ‘*a*’ and ‘*b*’ are, respectively, the larger and smaller diameter. At the end of the treatments, the mice in each group have been killed with CO_2_, 6 or 2 h after the last AS602868 or CPT-11 administration respectively. Tumours were removed, minced, put into liquid nitrogen or RNA later (Ambion, Huntingdon, UK), and stored at −80°C.

### Statistical analysis

Statistical significance of *in vivo* drug treatment effectiveness on tumour growth was calculated using ANOVA and the protective least significant difference using Fisher test. A probability of less than 0.05 was considered as significant. Additive or synergistic effect of drug combinations *in vitro* was evaluated using a non-constant ratio isobologram analysis with the CompuSyn software (ComboSyn Inc., New York, NY, USA). The combination index values were interpreted as follows: <1.0, synergism; 1.0, additive; and >1.0 antagonism.

### Cytotoxicity assay

Cytotoxic studies were carried out using an MTT assay (van de Loosdrecht *et al*, 1994), representing the percentage of growth inhibition induced by treatments. One thousand HT-29 cells were plated per well in 96-well plates with medium and various concentrations of AS602868±SN-38 for 5 days.

### EMSA and gel mobility shift assays

Nucleic extracts of HT-29 cells and tumours were prepared according to the method described by Dignam *et al* (1983). Briefly, 5 × 10^6^ cells were trypsinized, washed in PBS, and pelleted. Tumours were crushed in 500 *μ*l PBS and pelleted (1000 **g**, 5 min, 4°C). Cell pellets or tumours were then resuspended in 50–100 *μ*l of hypotonic buffer A. They were incubated for 10 min on ice, vortexed and centrifuged (10 000 **g**, 2 min, 4°C). Tumour supernatants (cytosolic extracts) were collected, cell pellets and tumours were suspended in 40–70 *μ*l of buffer B and centrifuged (13 000 **g**, 10 min, 4°C). Supernatants (nuclear extracts) were collected and diluted in 50–80 *μ*l of buffer C. EMSA were performed as described previously. For supershift assays, antibodies against p65 or p50 or rabbit IgG (4 *μ*g) were added 10 min before the labelled probe.

### Apoptosis and cell proliferation assays

Apoptosis was measured after a 5-day stimulation of HT-29 cells, plated as described for cytotoxicity assay using the cell death detection ELISA^plus^ Kit (Roche Diagnostics, Meylan, France). Cell proliferation was measured using the ELISA BrdU Kit from Roche Diagnostics. Assays were performed in triplicate following manufacturer's instructions.

### Western blot analysis

Total HT-29 cell extracts were prepared in lysis buffer as described previously (Frelin *et al*, 2003), incubated for 30 min on ice and centrifuged (10 000 **g**, 10 min, 4°C). HT-29 cell or tumour extracts were separated by sodium dodecyl sulphate-polyacrylamide gel electrophoresis on polyacrylamide gels and blotted on immobilon membranes (Millipore, Bedford, MA, USA). Primary antibodies were revealed with secondary peroxidase-conjugated antibody (DakoCytomation) followed by enhanced chemiluminescence detection (Amersham Pharmacia, Saclay, France).

### Reverse transcriptase-polymerase chain reaction

Total RNA from HT-29 cells or tumours was prepared in 2–4 ml of Trizol reagent (Invitrogen, Amsterdam, The Netherlands) according to Chomczynski and Sacchi (1987). A total of 1 *μ*g RNA was reverse transcripted using SuperScript II reverse transcriptase (Invitrogen) following manufacturer's instructions and resuspended in 12 *μ*l final volume. Two microlitre of the reverse-transcribed material were amplified by polymerase chain reaction (PCR) in 20 *μ*l reactions containing 0.5 *μ*l sense and antisense primers (Eurogentec, Angers, France); 0.6 *μ*l dNTP (20 mM); 2 *μ*l of Taq polymerase (New England Biolabs, Saint Quentin, France) at 5000 U *μ*l^−1^ of commercial buffer for a total of 30 cycles consisting of 94°C for 60 s, 55°C for 60s, and 70°C for 60 s. Ten microlitre amplification products were analysed by electrophoresis in ethidium bromide-stained agarose gels. Primer sequences are available upon request.

### Flow cytometric analyses

Cell cycle analysis was performed by quantifying DNA content using propidium iodide staining and analyzing by flow cytometry, as described previously (Vindelov *et al*, 1983).

### TUNEL analyses

Frozen tumour sections (7 *μ*m) were rehydrated in PBS, fixed for 20 min at room temperature using 3.7% formaldehyde and then permeabilized for 2 min in 0.1% Triton X-100 in 0.1% sodium citrate solution at 4°C. They were mounted in Fluoromount-G solution (Southern Biotechnology Associates Inc., Birmingham, AL, USA) and processed following the protocol described in the *In Situ* Cell Death Detection Kit (Roche Diagnostics). Analyses were performed using an LSM 510 confocal laser-scanning microscope (Carl Zeiss AG, Jena, Germany).

### Histology

Tumour sections (3 *μ*M) were incubated with an anti-Ki-67 (clone MIB-1) or anti-TNF*α* at room temperature for 30 min. After washing in PBS, a peroxydase-conjugated antibody was added for 30 min at room temperature and reaction developed using an AEC Kit (DakoCytomation). After haematoxylin counterstaining, slides were permanently mounted in an aqueous medium (Aquatex, Merck, Darmstadt, Germany) and analysed for the presence and the distribution of the immunostaining. For morphological studies, sections were stained with haematoxylin/eosin/safran (HES).

## RESULTS

### Inhibition of HT-29 cell viability *in vitro* by AS602868 in combination with SN-38

**Figure 1:**
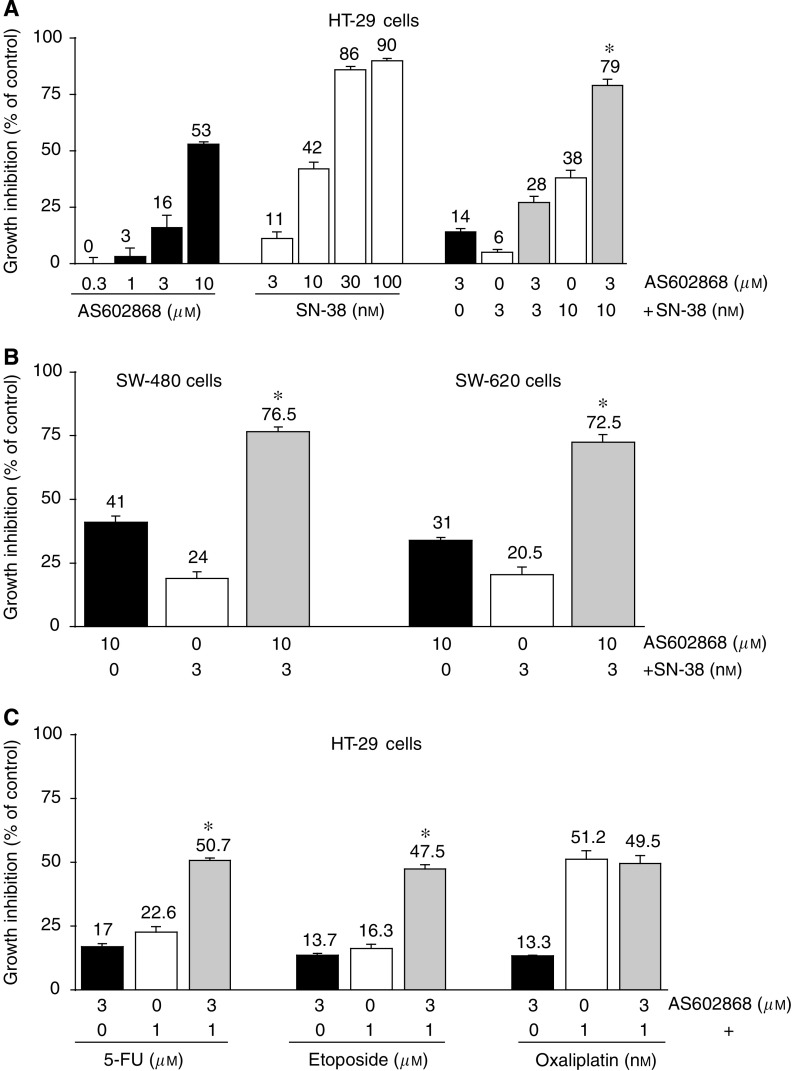
*In vitro* effect of AS602868 combined with SN-38 on cell viability. (**A**, **B**) HT-29 cells, SW-480, and SW-620 cells were incubated for 5 days with AS602868, SN-38, or both compounds simultaneously. (**C**) HT-29 cells were incubated for 5 days with 5-FU, etoposide, or oxaliplatin±AS602868 for 5 days. Cytotoxicity was evaluated using the MTT assay. Data are expressed as mean±s.d. of quadruplicates of one representative experiment out of 8 (**A**), 3 (**B**), and 3 (**C**). ^*^ indicates detection of the synergistic effect of AS602868 and SN-38, 5-FU, etoposide, or oxaliplatin on cell viability by using the non-constant ratio isobologram method.

After 5 days incubation, increasing concentrations of AS602868 or SN-38 resulted in a decrease in HT-29 cell viability (Figure 1A) in a dose-dependent manner, with a maximal effect for 10 *μ*M AS602868 and for 100 nM SN-38 (53 and 90% inhibition, respectively). As 3 and 10 ng ml^−1^ SN-38 are sub-lethal doses for HT-29 cells, these concentrations have been chosen in combined experiments. In the presence of both AS602868 (3 *μ*M) and SN-38, an additive (SN-38, 3 nM) or synergistic (SN-38, 10 nM) cytotoxic effect could be observed: 28 and 79% of cytotoxicity respectively. The IC_50_ for SN-38 on HT-29 cells estimated at ∼25 nM decreased to ∼10 nM in the presence of 3 *μ*M AS602868. A different sequencing order of the two drugs (AS602868 added 24 h before or after SN-38) had comparable effects on HT-29 cell viability to the simultaneous treatment (not shown). The potentiating effect of AS602868 on SN-38-mediated cytotoxicity was also observed on SW-480 and SW-620 tumour cells (Figure 1B). A synergistic cytotoxic effect was also observed when HT-29 cells were incubated with AS602868 plus 5-fluorouracil (5-FU) or etoposide but not in the presence of oxaliplatin (Figure 1C).

### Dose-dependent potentiation of CPT-11 antitumour activity by NF-*κ*B inhibition with AS602868 in xenograft experiments

**Figure 2:**
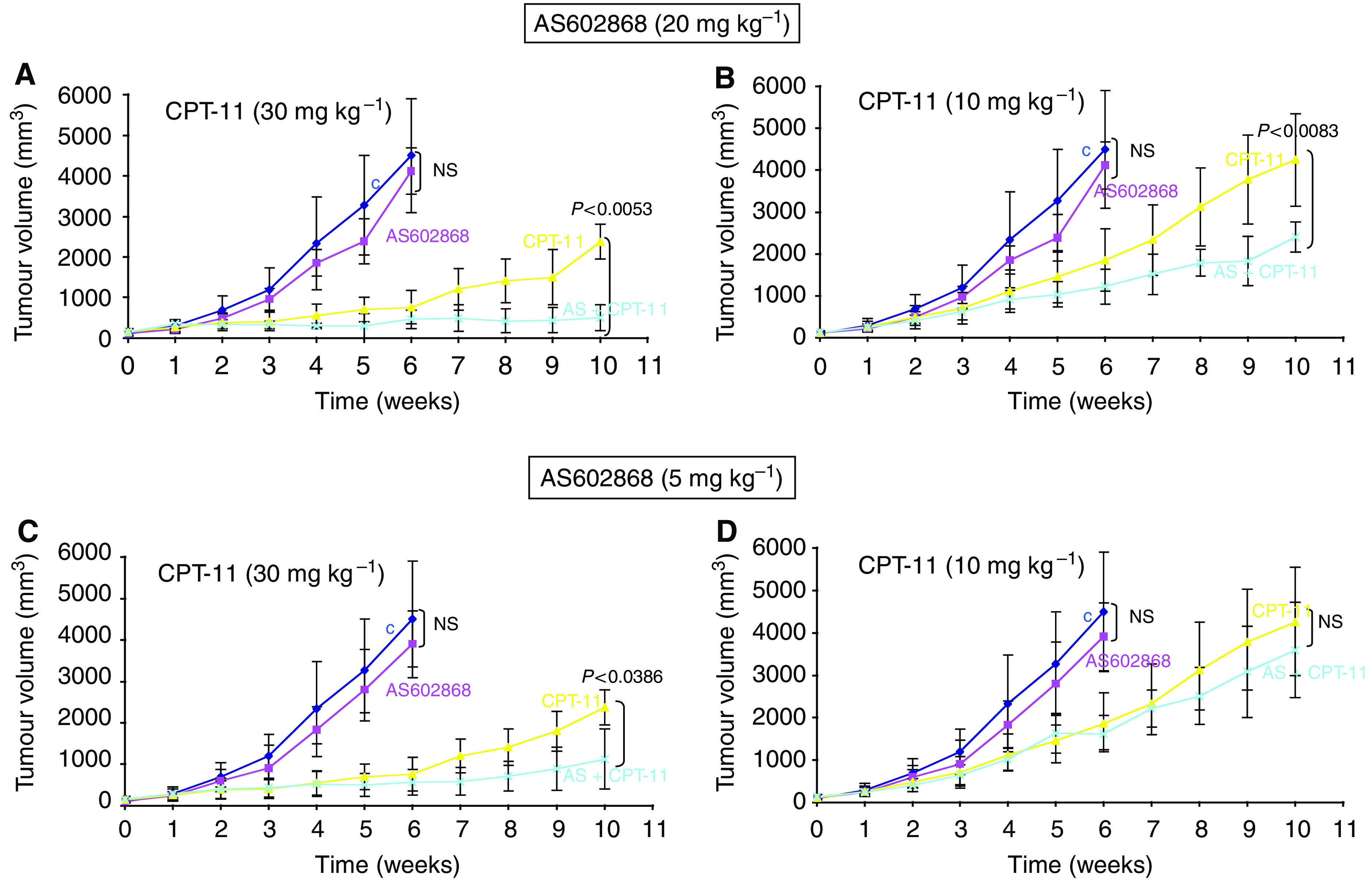
*In vivo* effect of AS602868 combined with CPT-11 on the development of s.c. HT-29 xenografts. (**A**–**D**) Evolution of HT-29 tumour volume. Nude mice received daily oral injections of AS602868, 5 days a week (
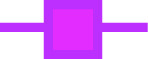
), and (
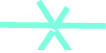
)/or (
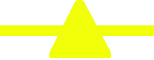
) CPT-11 i.p. injections twice a week, or vehicle buffer (
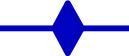
). Data are the mean±s.d. of tumour measurements using 5 mice/group and are representative of three other experiments. Statistically significant differences between control and AS602868-treated groups on the 6th week and between CPT-11 and CPT-11+AS602868-treated groups on the 10th week are indicated on each figure. NS, not significant.

Mice (5/group) were inoculated s.c. with HT-29 human colon tumour cells. Treatments started when the mean tumour volume was 150±44 mm^3^. Clinically achievable concentrations of AS602868 (5 or 20 mg kg^−1^) was administered either alone, or in combination with CPT-11 (10 or 30 mg kg^−1^) (Figure 2). No signs of visible toxicity (diarrhea, weight lost, apathy, hair or skin problems, etc.) were observed with any of the treatments. After 6 weeks, no significant differences in tumour size were observed between control mice and mice treated with 20 or 5 mg kg^−1^ AS602868 (Figure 2A–D respectively). These mice had to be killed for ethical reasons. After 10 weeks, CPT-11 (30 mg kg^−1^) strongly delayed tumour development (*P*<0.0001) (Figure 2A and C) and appeared 50% less efficient (*P*<0.0051) when administrated at 10 mg kg^−1^ (Figure 2B and D). Addition of AS602868 20 mg kg^−1^ (Figure 2A) or 5 mg kg^−1^ (Figure 2C) significantly potentiated the effect of 30 mg kg^−1^ CPT-11 (*P*<0.0053 and 0.0386 respectively). When CPT-11 was injected at 10 mg kg^−1^, AS602868 significantly improved (Figure 2B) CPT-11 antitumour effect at 20 mg kg^−1^ (*P*<0.0083) but not at 5 mg kg^−1^ (Figure 2D). The combination of CPT-11 (10 mg kg^−1^) plus AS602868 (20 mg kg^−1^) was as efficient as 30 mg kg^−1^ CPT-11. Potentiation of CPT-11 antitumour activity by AS602868 was also observed in two other colon xenograft models using SW-480 and SW-620 cell lines (not shown).

### Inhibition of CPT-11/SN-38-induced NF-*κ*B pathway activation by AS602868 *in vitro* and *in vivo*

**Figure 3:**
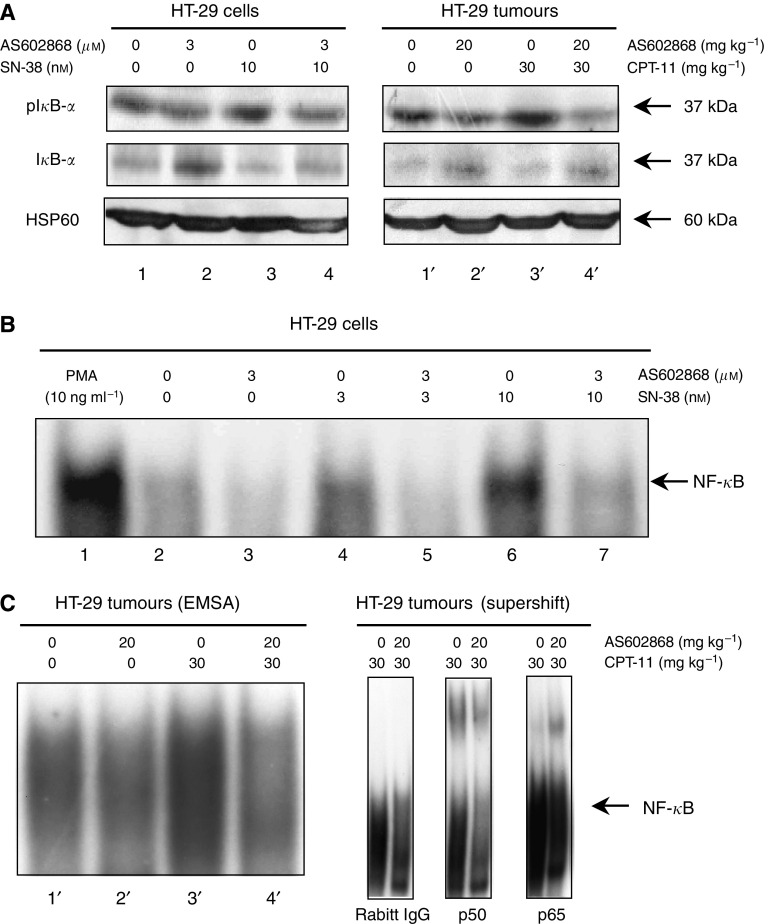
*In vitro* and *in vivo* effect of AS602868 combined with CPT-11/SN-38 on the NF-*κ*B pathway. (**A**) I*κ*B-*α* phosphorylation levels were studied by western blotting either on lysates of HT-29 cells that were stimulated 30 min with indicated concentrations of AS602868 and SN-38 or in tumours from mice treated as indicated. HSP60 was used as loading control. (**B**–**C**) NF-*κ*B activation was visualized by EMSA. HT-29 cells were treated with indicated concentrations of AS602868, 30 min before stimulation with SN-38 (3 and 10 nM) for 1 h or with PMA (10 ng ml^−1^) for 1 h as positive control. These results correspond to one representative experiment from 3. In supershift experiments, nuclear protein extracts of tumours from CPT-11 and AS602868±CPT-11-treated mice were incubated with anti-p50 and anti-p65 antibodies or rabbit IgG as negative control.

Western blotting experiments (Figure 3A) performed on HT-29 cells (left panel) or tumours (right panel) showed that I*κ*B-*α* phosphorylation was increased (upper row) while total levels were reduced (intermediate row) upon CPT-11 stimulation (lanes 3 and 3′ compared, respectively, to lanes 1 and 1′). I*κ*B-*α* phosphorylation was reduced and total I*κ*B-*α* enhanced when AS602868 was added (lanes 4 and 4′ compared, respectively, to lines 3 and 3′). As a control, we checked that no changes in Hsp60 levels were observed (lower row). Subsequent NF-*κ*B DNA-binding activity was then studied.

As shown by EMSA (Figure 3B), 1 h *in vitro* stimulation of HT-29 cells with SN-38 (3 and 10 nM) induced NF-*κ*B activation in a dose-dependent manner (lanes 4 and 6 compared to lane 2). This was dramatically decreased after incubation with AS602868 (3 *μ*M) (lanes 4 and 6 compared, respectively, to lanes 5 and 7). AS602868 also inhibited the weak constitutive activity of NF-*κ*B observed in HT-29 cells (lane 3 compared to 2). SN38 had less of an affect on NF-*κ*B activation compared to PMA (lane 6 *vs* 1). Similar results were observed in tumours (Figure 3C, left panel). The specificity of the NF-*κ*B DNA-binding activity was demonstrated by competitive inhibition in the presence of a 100 ng excess of unlabelled probe (not shown). When EMSAs were performed in the presence of anti-p52, c-Rel, Rel-B, p50, and p65 antibodies in tumour extracts from mice treated with CPT-11 or CPT-11 plus AS602868, supershifts were obtained only in the presence of anti-p50 and anti-p65 antibodies (Figure 3C, right panel). Therefore, CPT-11 appears to mobilize classical p50–p65 NF-*κ*B complexes.

### Induction and potentiation of SN-38-mediated apoptosis of HT-29 colon tumour cells by AS602868

**Figure 4:**
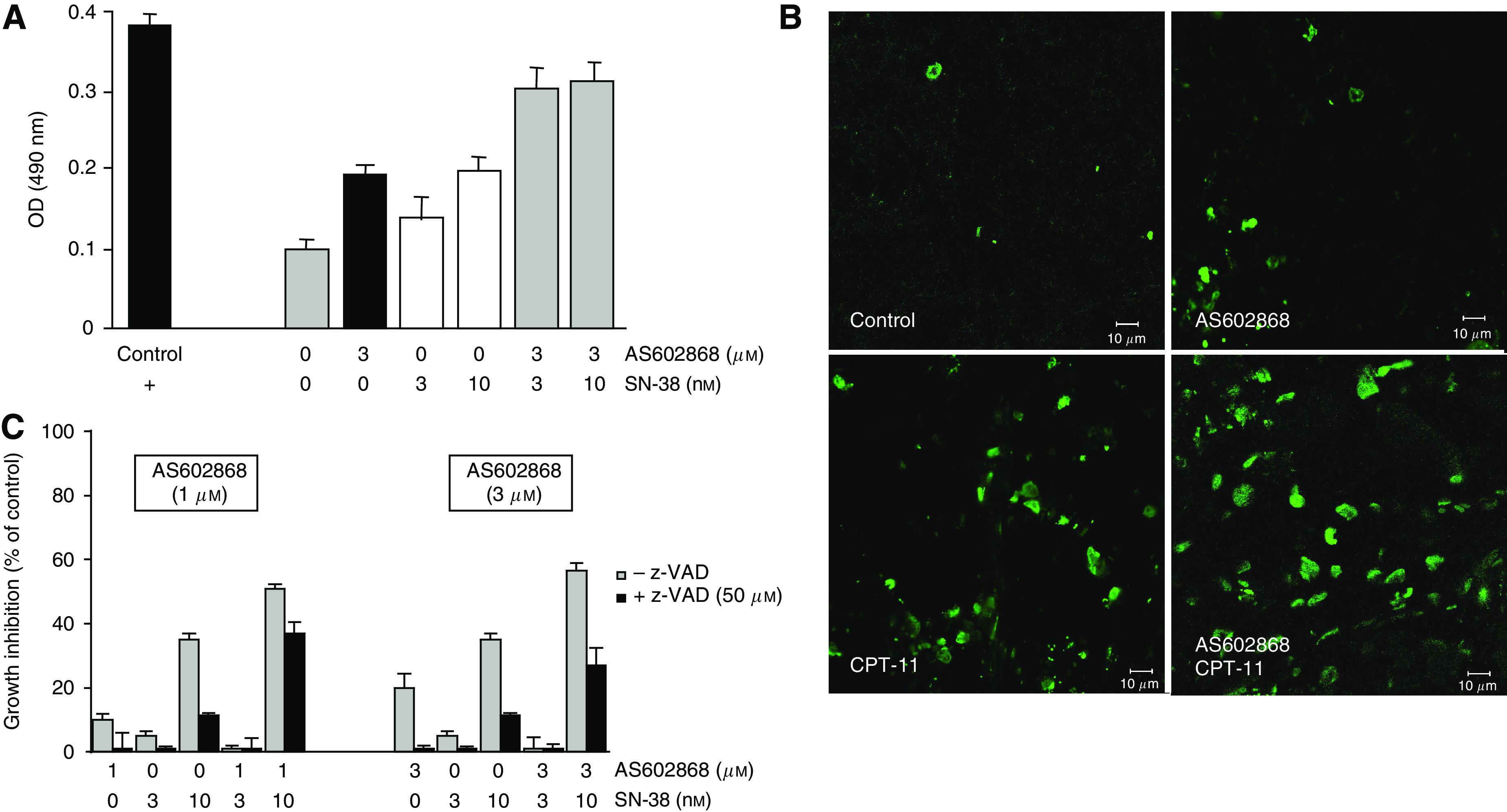
*In vitro* and *in vivo* effect of AS602868 combined with CPT-11/SN-38 on apoptosis. (**A**) Apoptosis was measured by ELISA. HT-29 cells were incubated for 5 days with AS602868±SN-38 at indicated concentrations. Data are expressed as mean OD±s.d. of duplicates of one representative experiment from 4. The positive control is a DNA–histone complex included in the kit. (**B**) Tumours were removed from mice treated with AS602868 vehicle (control group), with AS602868 (20 mg kg^−1^) alone or combined with CPT-11 (30 mg kg^−1^). Then, they were minced, put into liquid nitrogen, and stored at −80°C. Frozen tumour sections (7 *μ*m) were mounted in Fluoromount-G solution and processed following the protocol described in the *In situ* Cell Death Detection Kit (Roche Diagnostics). Analyses were performed using an LSM 510 confocal laser-scanning microscope with an oil objective × 40. (**C**) HT-29 cells were treated with AS602868±SN-38±z-VAD^fmk^ (50 *μ*M) for 5 days. Cell viability was evaluated using the MTT assay. Data are expressed as means±s.d. of quadruplicates of one representative experiment from 3.

Quantification by ELISA (Figure 4A) of mono- and oligonucleosomes showed that AS602868 (3 *μ*M) or SN-38 (3 and 10 nM) alone induced HT-29 cell apoptosis. A higher effect was produced by combining the two drugs: 3 *μ*M AS602868 (two-fold medium OD), 3 and 10 nM SN-38 (1.5 and 2-fold medium OD, respectively), compared to (three-fold medium OD) obtained with 3 *μ*M AS602868+3 nM SN-38 and (3.2-fold) with 3 *μ*M AS602868+10 nM SN-38. Similar results were obtained *in vivo* (Figure 4B). TUNEL experiments revealed nearly no apotosis in tumours from mice of the control group, and an increasing level in tumours from mice treated with AS602868, CPT-11, and with the combined treatment.

Figure 4C indicates that the cytotoxic effect of AS602868 and SN-38 is only partly caspase-dependent since z-VAD^fmk^ (a pan caspase inhibitor) could only prevent the decrease in viability by 40% on average. Thus, the induction of apoptosis cannot completely explain AS602868 and SN-38 cytotoxic effect.

### Induction and potentiation of SN-38-mediated pro-caspase cleavage in HT-29 cells and tumours by AS602868

**Figure 5:**
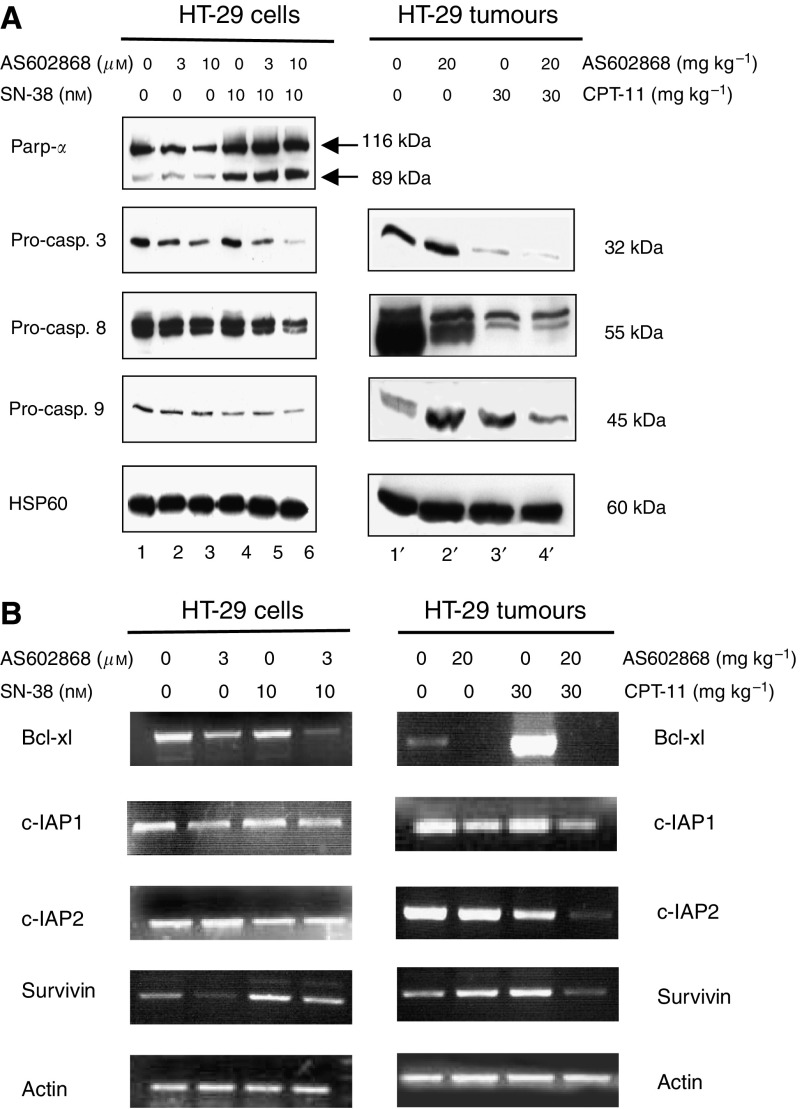
Effect of AS602868 combined with CPT-11/SN-38 on pro-caspase cleavage and anti-apoptotic gene expression in HT-29 cells and tumours. (**A**) Cleavage of Parp-*α* and pro-caspases was demonstrated by western blotting either on lysates of HT-29 cells incubated for 72 h with indicated concentrations of AS602868±SN-38 or in cytoplasmic protein extracts of HT-29 tumours from mice treated as indicated. HSP60 was used as loading control. (**B**) Anti-apoptotic gene expression was studied by RT-PCR analysis on RNA extracted either from HT-29 cells stimulated for 72 h with indicated concentrations of AS602868±SN-38 or from HT-29 tumours. Experiments were performed on 1 *μ*g RNA and amplification of cDNA was of 30 cycles. Actin was used as an invariant control.

In HT-29 cells, AS602868 induced a dose-dependent cleavage of pro-caspases 3 and 8, had a slight effect on pro-caspase 9, and no effect on the caspase substrate Parp-*α* (Figure 5A: lanes 2 and 3). SN-38 (10 nM) induced cleavage of Parp-*α* and pro-caspase 9 and had a weak effect on pro-caspase 8 (lanes 4 and 5). Combining AS602868 with SN-38 resulted in a higher proteolysis of pro-capases 3, 8, and 9 and of Parp-*α* (lane 6). Overall, results were comparable to those found in tumour extracts (lanes 1′–4′), but for unknown reasons it has not been possible to detect Parp-*α* in tumour extracts, whatever the protocol used. Thus, *in vivo* and *in vitro* inhibition of NF-*κ*B activity using AS602868 allowed the potentiation of the processing of pro-caspases 3, 8, and 9.

### AS602868 inhibits expression of NF-*κ*B anti-apoptotic target genes *in vitro* and *in vivo*

In HT-29 cells, AS602868 decreased the expression of Bcl-xl, c-IAP1, and survivin but not that of c-IAP2 (Figure 5B: lane 2 compared to lane 1). SN-38 had nearly no effect on the expression of theses genes except a slight increase in survivin gene expression (lane 3). However, combining AS602868 with SN-38 further decreased Bcl-xl expression and to a lesser extent that of c-IAP1 and survivin (lane 3 compared to lane 4). In tumours (Figure 5B: lanes 1′–4′), each compound alone had minor effect on expression of these genes except a strong increase in Bcl-xl expression was observed after CPT-11 treatment. The combination therapy dramatically decreased CPT-11-induced Bcl-xl expression, below baseline level. The levels of c-IAP1 and 2 and survivin were also decreased (lane 4′).

### Inhibition of necrosis, tumour cell proliferation, and cell cycle progression in HT-29 cells and tumours by AS602868

**Figure 6:**
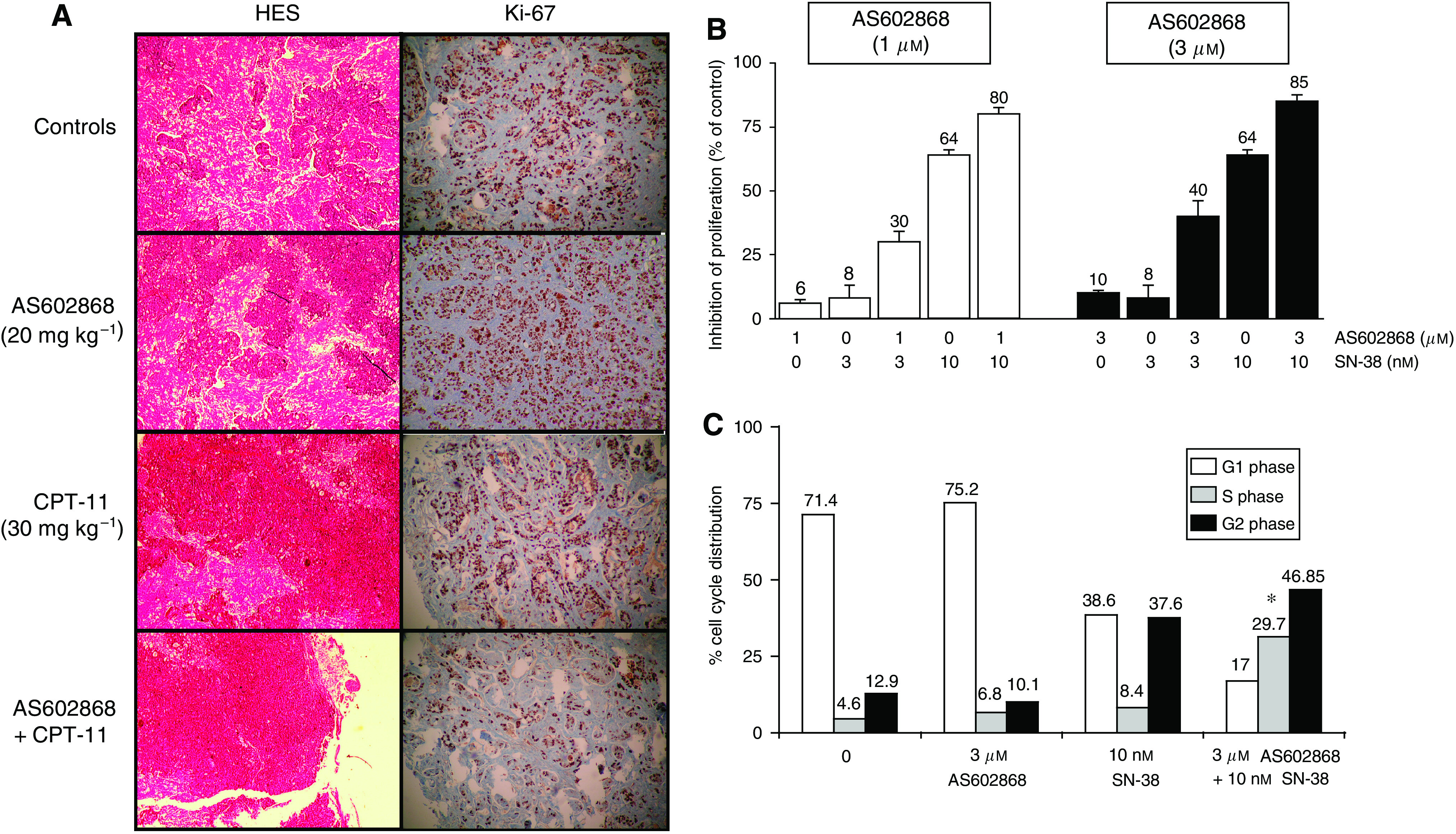
Effect of AS602868 combined with CPT-11 on necrosis, cell proliferation, and cell cycle progression in HT-29 cells and tumours. (**A**) Histological examination of HT-29 tumour xenografts after 6 (control and AS602868 groups) or 10 weeks of treatment (CPT-11 and CPT-11+AS60286 groups). Tumour sections were stained with HES (necrosis) or anti-Ki-67 (proliferation staining). Magnification: × 320. (**B**) Quantification of AS602868±SN-38 effect on HT-29 cancer cell proliferation by ELISA based on BrdU incorporation in DNA. (**C**) Measure of AS602868±SN-38 effect on cell cycle progression by flow cytometric analyses using propidium iodide staining. Cells were stimulated for 72 h after adhesion. ^*^ indicates detection of the synergistic effect of AS602868 and SN-38 by using the non-constant ratio isobologram method.

Histological examination of HT-29 xenografts (Figure 6A) revealed an extensive necrosis in tumours (HES staining) from control and AS602868 groups that decreased by two-fold in tumours from the CPT-11 group and by four-fold in tumours from the AS602868+CPT-11 group. Ki-67 staining revealed a two-fold decrease in tumour cell proliferation in the AS602868+CPT-11 group compared to the other groups. BrdU incorporation (Figure 6B) showed that the combination of suboptimal doses of AS602868 (1 and 3 *μ*M) and SN-38 (3 and 10 nM) had additive effect to inhibit cell proliferation with a maximum of 85% inhibition with 3 *μ*M AS602868 plus 10 nM SN-38.

Cell cycle analysis (Figure 6C) revealed that AS602868 or SN-38 alone induced a slight increase in the number of cells in S phase (6.8 and 8.4, respectively, compared to 4.6%). SN-38 also strongly blocked HT-29 cells in G2/M (37.6 compared to 12.9%). Combining AS602868 (3 *μ*M) with SN-38 (10 nM) resulted in a synergistic blockade in S phase (29.7%) and an additive blockade in G2/M phase (46.9%). The number of cells in S phase was increased by 6.5-fold compared with 1.5- or 1.8-fold with AS602868 or SN-38 alone, respectively.

### Increase of TNF*α* concentration by CPT-11 in HT-29 tumours and induction of TNF*α* apoptosis potential in HT-29 cells by AS602868

**Figure 7:**
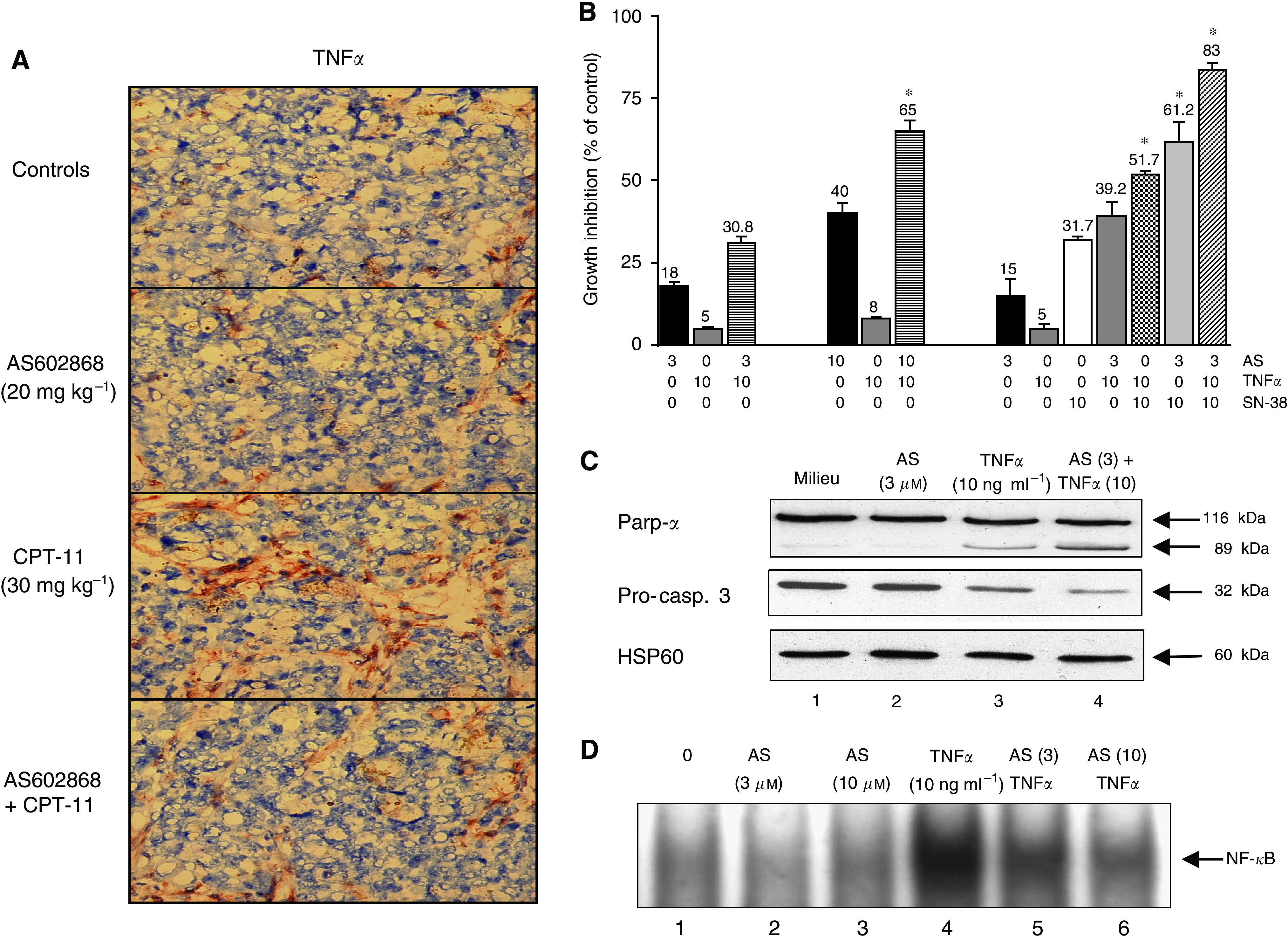
Effect of AS602868 combined with CPT-11 on TNF*α* intratumoural concentration and induction of TNF*α* apoptosis potential in HT-29 cells by AS602868. (**A**) Histological examination of HT-29 tumour xenografts after 6 (control and AS602868 groups) or 10 weeks of treatment (CPT-11 and CPT-11+AS60286 groups). Tumour sections were stained with an anti-TNF*α* antibody. Magnification: × 320. (**B**) HT-29 cells were incubated for 5 days with AS602868, TNF*α* or both molecules simultaneously or SN-38, TNF*α* or both molecules or AS602868, TNF*α* and SN-38 together. Cytotoxicity was evaluated using the MTT assay. Data are expressed as means±s.d. of quadruplicates of one representative experiment from 3. ^*^ indicates detection of the synergistic effects of AS602868±TNF*α*±SN-38 on cell viability by using the non-constant ratio isobologram method. (**C**) Cleavage of Parp-*α* and pro-caspase 3 was demonstrated by western blotting on lysates of HT-29 cells incubated for 72 h with indicated concentrations of AS602868±SN-38. HSP60 was used as loading control. (**D**) NF-*κ*B activation was visualized by EMSA. HT-29 cells were treated with indicated concentrations of AS602868, 30 min before stimulation with TNF*α* (10 ng ml^−1^) for 1 h. These results correspond to one representative experiment from 3.

Histological examination of HT-29 xenografts (Figure 7A) revealed the presence of low concentrations of TNF*α* in tumours from control mice and AS602868-treated groups. The CPT-11 treatment induced a three-fold increase in intratumoural TNF*α* concentration, which was decreased to two-fold upon co-treatment with AS602868.

NF-*κ*B is known to inhibit apoptosis induced by TNF*α*. Alone, TNF*α* (10 ng ml^−1^) induced a minimal (5%) decrease in viability of HT-29 cells (Figure 7B). However, addition of AS602868 at 3 *μ*M (18% cytotoxicity) had an additive effect (30.8%) while at 10 *μ*M (40% toxicity) it produced a synergistic effect with TNF*α* (65%). A comparable synergistic effect was observed when 10 nM SN-38 was added to 10 ng ml^−1^ TNF*α* (31.7 compared to 51.7% cytotoxicity) or in the presence of the three compounds (83% of cytotoxicity). TNF*α* induced a small decrease in Parp-*α* and pro-caspase 3 but not in HSP60 levels (Figure 7C, lane 3). AS602868, which had no effect by itself (lane 2), increased the cleavage of Parp-*α* and pro-caspase 3 by TNF*α* (lane 4 *vs* lane 3). As showed by EMSA (Figure 7D), AS602868 inhibited TNF*α*-induced NF-*κ*B activation in a dose-dependent manner (lanes 5, 6 compared to 4).

## DISCUSSION

NF-*κ*B activation by antineoplastic drugs is one of the mechanisms for tumour resistance to chemotherapy (Baldwin, 2001; Nakanishi and Toi, 2005). In the present study, we show that pharmacological inhibition of the NF-*κ*B pathway by the IKK2 inhibitor AS602868 potentiates the antitumoural effect of CPT-11 *in vivo* and that of its active metabolite SN-38 *in vitro*. Interestingly, in xenograft experiments, the combined treatment allowed a three-fold decrease in CPT-11 concentration without any loss in efficiency. Inhibition of NF-*κ*B was also observed to reveal an apoptotic action of TNF*α* on HT-29 cells, whose intratumoural concentrations were increased upon CPT-11 treatment. Furthermore, in agreement with previous data (Wang *et al*, 2003; Voboril *et al*, 2004), inhibition of NF-*κ*B also augmented sensitivity of HT-29 tumour cells to 5-FU, the most common antimetabolite used for the treatment of CRC and other types of solid tumours.

In haematopoietic malignancies, inhibition of abnormal constitutive NF-*κ*B activity frequently results in the death of leukaemic cells (Frelin *et al*, 2005; Garcia *et al*, 2005). In solid tumours like CRC, however, a combined therapy with antineoplastic drugs appears necessary to get similar results. Inhibition of NF-*κ*B through the intratumoural adenoviral delivery of a super repressor form of I*κ*B*α*, in combination with CPT-11, led to a considerable growth suppression of Lovo colon tumours associated with an enhanced apoptotic response (Wang *et al*, 1999). In the same tumour model, i.v. administration of the proteasome inhibitor PS-341 prior to CPT-11 inhibited NF-*κ*B activation, resulting in a marked decrease in tumour size (Cusack *et al*, 2001). The level of apoptosis reached 80–90% in the group receiving combined treatments compared with 10% in tumours treated with single agents. Recently, the new proteasome inhibitor NPI-0052 has also been demonstrated to significantly improve the tumouricidal response of chemotherapy when orally administered in Lovo xenograft-bearing mice, by increasing apoptosis and shifting cells towards G2 cell cycle arrest (Cusack *et al*, 2006). NPI-0052 effects resulted in a 1.8-fold increase in response to CPT-11, 5-FU, and leucovorin triple drug combination; a 1.5-fold increase in response to the oxaliplatin, 5-FU, and leucovorin triple drug combination; and a 2.3-fold greater response to the CPT-11, 5-FU, leucovorin, and Avastin regimen. Reduction of endogenous p65 by siRNA treatment in HCT-116 colon cancer cells significantly impaired CPT-11-mediated NF-*κ*B activation, enhanced apoptosis, decreased colony formation in soft agar and when administered *in vivo*, reduced HCT-116 tumour formation in the presence but not in the absence of CPT-11 (Guo *et al*, 2004). Our results appear consistent with these studies and show that the inhibition of NF-*κ*B by AS602868, easy to use, can potentiate chemotherapeutic drug efficiency in CRC.

The potentiating effect of AS602868 on HT-29 tumours appears to be partly due to enhanced CPT-11-induced apoptosis. AS602868 alone had moderate effects on apoptosis by itself but activation of caspases 3, 8, and 9 could be easily detected when the inhibitor was combined with CPT-11 or SN-38. Thus, the two cellular apoptotic pathways appeared mobilized by combined treatments. A decrease in the transcription of several anti-apoptotic NF-*κ*B target genes such as Bcl-xl, survivin, and to a lesser extent c-IAP1 and c-IAP2 was observed which could reflect an overall decrease in survival influences preceding caspase activation. AS602868 has been previously shown to induce apoptosis of human acute myeloid leukaemia cells (Frelin *et al*, 2005), effects that were associated with disruption of the mitochondrial potential and by activation of pro-caspases 9 and 3. It has been demonstrated that p65 siRNA enhanced CPT-11-mediated apoptosis by increasing caspase 3 activity and lowering c-IAP 1 and c-IAP 2 protein levels (Guo *et al*, 2004). Moreover, antisense Bcl-xl downregulation in HCT-11 colon cancer cells switched the response to topoisomerase 1 inhibition from senescence to apoptosis, enhancing global cytotoxicity (Hayward *et al*, 2003). These results suggest that NF-*κ*B likely supports cell survival by different means depending on cell types and/or underlying oncogenic mechanisms.

The level of necrosis in HT-29 tumours was inversely proportional to tumour size indicating that necrosis cannot account for AS602868/CPT-11 effects. On the other hand, autophagy could be a possible mechanism of action for AS602868 since it has recently been shown that a direct cross-talk exists between autophagy and NF-*κ*B reviewed in Xiao (2007). The combined treatment also reduced tumour cell proliferation. In agreement with previous data (Ohwada *et al*, 1996; Xu and Villalona-Calero, 2002), we found that SN-38 alone markedly inhibited HT-29 cell proliferation by arresting cells mainly at the G2/M phase and, to a lesser extent, at the S phase. AS602868 for its part only induced a modest arrest at the S phase, but in combination with SN38 increased the number of cells in both S and G2/M phases. Thus, the enhanced antitumour effect of the combined therapies could also be explained by the ability of AS602868 to increase the number of cells in S phase as these cells are 100–1000 times more sensitive to CPT-11 (Li *et al*, 1972).

Invalidation of the genes coding for IKK2, NEMO, or RelA resulted in early embryo death from TNF-dependent liver apoptosis demonstrating the important anti-apoptotic functions of NF-*κ*B (Karin and Lin, 2002). Moreover, the expression of a non-phosphorylatable form of the IkB-*α* subunit, which acts as a super-repressor of NF-*κ*B activation, increased apoptotic responses in various cell lines stimulated by TNF*α* (Van Antwerp *et al*, 1998). As CPT-11 treatment resulted in an increase in TNF*α* intratumoural concentration and as AS602868 revealed the apoptotic potential of TNF*α* in HT-29 colon cancer cells as well as in Jurkat leukaemic cells (Frelin *et al*, 2003), this mechanism could also be involved in AS602868/CPT-11 antitumoural effect. Furthermore, the suppressor protein p53 is mutated in HT-29 cells (Goldberg *et al*, 1996; Gobert *et al*, 1999) and recent data showed that p53 mutations may promote cancer progression by augmenting NF-*κ*B activation in the context of chronic inflammation (Weisz *et al*, 2007).

To our knowledge, none of the many NF-*κ*B signaling inhibitors described so far (Gilmore and Herscovitch, 2006) have been shown *in vivo* to increase chemotherapy efficiency in CRC. Taken together, our results provide a rationale for using the IKK2 inhibitor AS602868 combined with CPT-11 as a promising therapeutic strategy for clinical testing in CPT-11 refractory CRC and probably other solid tumours. Of course the toxicity/efficacy ratio will be a crucial factor for the therapeutical use of AS602868 molecule. However, preliminary results warrant that clinical trials will be performed.
